# Synergistic modulation of cyclobutane pyrimidine dimer photoproduct formation and deamination at a T^m^CG site over a full helical DNA turn in a nucleosome core particle

**DOI:** 10.1093/nar/gku1049

**Published:** 2014-11-11

**Authors:** Qian Song, Vincent J. Cannistraro, John-Stephen Taylor

**Affiliations:** Department of Chemistry, Washington University, St. Louis, MO 63130, USA

## Abstract

Sunlight-induced C to T mutation hotspots in skin cancers occur primarily at methylated CpG sites that coincide with sites of UV-induced cyclobutane pyrimidine dimer (CPD) formation. The C or 5-methyl-C in CPDs are not stable and deaminate to U and T, respectively, which leads to the insertion of A by DNA polymerase η and defines a probable mechanism for the origin of UV-induced C to T mutations. We have now determined the photoproduct formation and deamination rates for 10 consecutive T=^m^CG CPDs over a full helical turn at the dyad axis of a nucleosome and find that whereas photoproduct formation and deamination is greatly inhibited for the CPDs closest to the histone surface, it is greatly enhanced for the outermost CPDs. Replacing the G in a T=^m^CG CPD with A greatly decreased the deamination rate. These results show that rotational position and flanking sequence in a nucleosome can significantly and synergistically modulate CPD formation and deamination that contribute to C to T mutations associated with skin cancer induction and may have influenced the evolution of the human genome.

## INTRODUCTION

Nucleosomes are the primary structural unit of chromatin in eukaryotic cells (1). Nucleosomes contain about 147 bp of duplex DNA that wraps 1.7 times around a histone octamer composed of two H2A, H2B, H3 and H4 histones (2). Ultraviolet (UV) light preferentially induces the formation of cyclobutane pyrimidine dimer (CPD) DNA photoproducts in nucleosomes at sites where the phosphodiester backbone is positioned away from the histone core surface and DNA bending is toward the major groove (3,4). This preference is also seen for bent DNA that is not in contact with a protein (5) and DNA in nucleosomes unfolded at very low ionic strengths (6). This has been attributed to the greater degree of rotational freedom in the phosphate backbone, making it more likely that adjacent pyrimidines adopt a photoreactive conformation (3,7,8). CPDs not only form preferentially at outside positions in bent DNA but can also induce rotational orientations in nucleosomes. When DNA containing randomly distributed CPDs is assembled into nucleosomes, the CPDs favor positions away from the surface (9), which is consistent with the 30° bending that they make toward the major groove of DNA (10).

**Figure 1.**
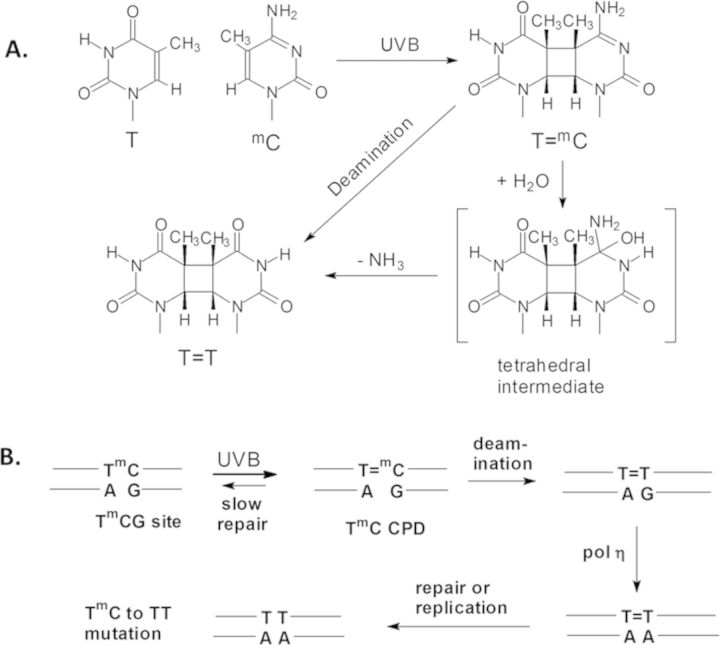
Deamination-bypass mechanism for UV-induced C to T mutations. (A) Formation of a CPD at a T^m^C site and its deamination. (B) Deamination bypass pathway for the formation of UV-induced C to T mutation at T^m^CG sites in which polymerase η inserts an A opposite the T resulting from deamination of the 5-methylC in the CPD formed by UV light.

Sunlight-induced C to T mutation hotspots in skin cancers occur primarily at methylated PyCG sites that coincide with sites of UV-induced CPD formation (11,12). The C's and 5-methyl-C's in the initially formed CPDs are not stable and deaminate within hours to days to U's and T's (13), which have been shown to direct the insertion of A's by DNA polymerase η (14). Thus, deamination followed by trans-lesion synthesis of PyCG and Py^m^CG CPDs would therefore explain the origin of UV-induced C to T and CC to TT signature mutations (deamination-bypass mechanism) (Figure 1). CPDs at TCG sites deaminate about 20-fold faster than at CCG sites (13), which would explain the much higher C→T mutation frequencies at TCG sites in both UVB and UVA irradiated mice (15,16) and the predominance of a T^m^CG to TTG mutations in the p53 gene of UVB-induced mouse tumors (17). It was recently discovered that nucleosomes can dramatically enhance or retard deamination at T^m^CG sites, with an outside facing CPD deaminating 45-fold faster than one facing inside (18). In that study, it was not established whether the two sites studied corresponded to the two extremes of the deamination rates, or how deamination rates varied with other rotational positions and flanking sequence. In this paper, we examined photoproduct formation and deamination for 10 consecutive T=^m^CG CPDs located at the nucleosome dyad axis as well as two T=^m^CA sites corresponding to the two most extreme rates of deamination. We found that the deamination rate depends on both rotational position and flanking sequence that could contribute to the variation in UV-induced C to T mutation frequencies and the origin of C to T mutation hotspots.

## MATERIALS AND METHODS

### DNA substrates

Oligodeoxynucleotides (ODNs) with or without 5′-terminal phosphates were purchased from Integrated DNA Technologies and purified by denaturing gel electrophoresis prior to ligation with T4 DNA ligase and adenosine triphosphate in the presence of complementary 20-mer ligation scaffolds. Substrates for the deamination reactions were prepared by 5′-^32^P end labeling the ODNs containing the 5′-terminal 5-methylC prior to ligation. Substrates for hydroxyl radical footprinting and Maxam–Gilbert G reactions were 5′-end labeled following ligation. The 147-mer single-strand products were purified by denaturing polyacrylamide gel electrophoresis (PAGE). Complementary 147-mers were then annealed to form the 147 base pair duplexes that were subsequently purified by native PAGE.

### Nucleosome reconstitution

Nucleosome core particles were isolated and purified from chicken erythrocytes following a detailed procedure provided by Dr. Michael Smerdon. An equimolar ratio mixture of the ten 147-mer DNA duplexes ds-1 to ds-10, and also individual 147-mer DNA duplexes (NCP-1 to NCP-10) were reconstituted with the chicken nucleosome core particles by slow dialysis from high to low salt. Briefly, 10 nM 147-mer duplexes were incubated with increasing amounts of nucleosome core particles (from 100 to 1000 nM) in a total volume of 500 μl, containing 2 M NaCl, 10 mM Tris-HCl, 5 mM DTT and 5 mM ethylenediaminetetraacetic acid (EDTA) at pH 7.5 at room temperature for 2 h, and then dialyzed against 50 mM NaCl, 10 mM Tris-HCl, 1 mM DTT, pH 7.5, at 4°C, overnight. The reconstituted particles were then equilibrated at 55°C for 2 h to fix the nucleosome phasing. The reconstituted particles were assayed by native PAGE (6% acrylamide, 0.2% bisacrylamide in TBE) and the ratio of nucleosome-bound DNA to free DNA was quantified by the Quantity One software.

### Hydroxyl radical foot-printing and dimethylsulfate mapping

Hydroxyl radical foot-printing was performed essentially as described previously (19). A 15 μl aliquot of 10 mM sodium ascorbate, a 15 μl aliquot containing 1 mM Fe(NH_4_)_2_(SO_4_)_2_·6H_2_O and 2 mM EDTA and 15 μl of a 0.12% (w/w) H_2_O_2_ solution were premixed and added within 5 s to 105 μl of the nucleosome separately reconstituted with the equimolar mixture of the 5′-^32^P-end labeled 147-mer DNA duplexes ds-1 to ds-10. The reactions were incubated for 120 s at room temperature and stopped by the addition of 16 μl of 50% (v/v) glycerol and 4 μl of 500 mM EDTA. The samples were electrophoresed on a native gel (6% acrylamide, 0.2% bisacrylamide in TBE), and the nucleosome bands electroeluted in TBE. The histone proteins were extracted with phenol:chloroform:isopropyl alcohol 25:24:1, and the DNA precipitated with ethanol. The protein-free ds-control DNA was treated in a similar way, except that the reaction was quenched with a solution containing 1 M sodium acetate, 120 mM thiourea, 300 μg/ml salmon sperm DNA and 60 mM EDTA at pH 6.5 and then ethanol-precipitated. A Maxam–Gilbert G sequencing reaction was carried out on each individual 5′-^32^P-labeled 147-mer DNA duplex ds-1 to ds-10 in 50 mM cacodylate, 50 mM NaCl, 5 mM EDTA in the presence of 10 nM ds-control. For a 50 μl reaction, 0.5 μl of dimethyl sulfate was added to initiate the reaction, and 10 μl aliquots were removed over time and quenched by the addition of 50 μl of 1.5 M sodium acetate, 1 M mercaptoethanol and 50 μg of denatured salmon sperm DNA. The samples were ethanol-precipitated twice, and the resulting pellets were vacuum-dried and then taken up in 100 μl of 1 M piperidine. The samples were then heated at 90°C in 1 M piperidine for 30 min and then evaporated to dryness at 60°C.

### Deamination rate assay

The deamination rates were determined by adapting our previously described method (13,18). The free and nucleosome bound DNA substrates were irradiated with 302 nm UVB light at 4°C for 1 h and then adjusted to pH 7.2 with Mes buffer (0.5 M) and incubated at 37°C. Aliquots (10 μl) were removed at various times and quickly frozen on dry ice before storing at −80°C. One aliquot was adjusted to pH 6.5 with Mes buffer (0.5 M) and heated at 67°C overnight to complete deamination. The aliquots were then warmed to room temperature, extracted with phenol:chloroform:isopropyl alcohol 25:24:1, ethanol-precipitated, redissolved in buffer containing 10 mM Tris-HCl, pH 7.5, 50 mM NaCl, 5 mM DTT and then photoreverted with photolyase and 365 nm light for 1 h. After photoreversion, the aliquots were treated with nuclease P1 to degrade the DNA to mononucleotides containing either ^32^P-d^m^C or ^32^P-dT, depending on the extent of deamination, and separated by stepwise gel electrophoresis. In the first step, the digested samples were electrophoresed on a 7 M urea TBE gel to separate ^32^P-d^m^C and ^32^P-dT, which co-migrate, from partially digested material. In the second step the gel surrounding the mononucleotide band was removed and a second gel containing 25 mM citric acid, pH 3.5 and 7 M urea was poured around the remaining rectangular gel slice. Electrophoresis in this gel separates ^32^P-d^m^C from ^32^P-dT with the ^32^P-dT migrating fastest. The deamination rate constants were then obtained from the slope of a linear least squares fit of the log of the fraction of T^m^C CPD versus deamination time. The fraction of T^m^C CPD was calculated as 1− (T/(T + ^m^C))/(T_∞_/(T_∞_ + ^m^C)), where T_∞_/(T_∞_ + ^m^C_∞_) is the fraction T/(T + ^m^C) in the fully deaminated sample. The yield of CPD photoproduct was calculated as the T_∞_/(T_∞_ + ^m^C_∞_).

## RESULTS

### Design and synthesis of DNA substrates

**Figure 2.**
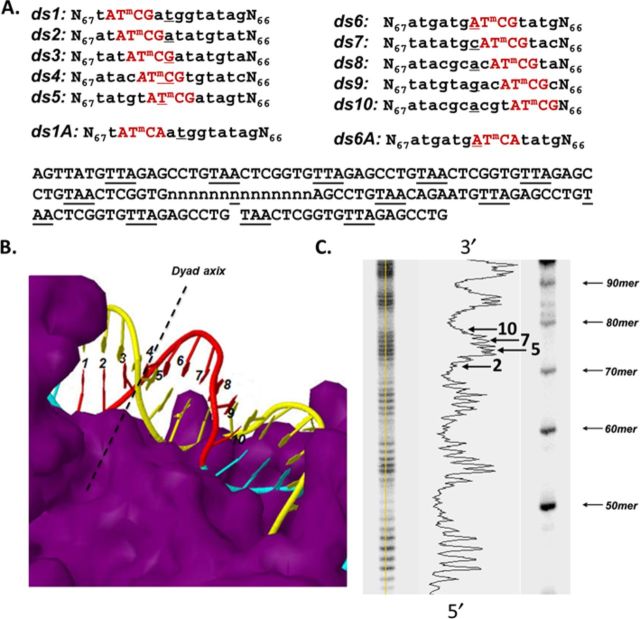
System for studying effect of nucleosome structure on deamination of T^m^CG CPDs. (A) Ten 147 base pair duplexes ds1–10 were constructed that would position a methyl-C (within AT^m^CG) through 10 consecutive rotational positions located at the nucleosome dyad. (B) Rotational positions of the ^m^C of the T^m^CG sites in the nucleosome. (C) Hydroxyl radical footprinting of nucleosomes reconstituted with equal molar amounts of the ten 147-mer duplexes. Cleavage intensities are shown on the right. Arrows indicate the site of ^m^C for the numbered substrate.

The purpose of this study was to determine the deamination rates for AT=^m^CG CPD sites spanning a full helical turn of DNA in a nucleosome in order to gain insight about the effect of chromatin structure on the frequency of mutations arising from a deamination-bypass mechanism. Nucleosome core particles were designed to contain a T=^m^CG CPD in each of 10 sequential rotational positions at the nucleosome dyad axis. The CPD sites were positioned using TG bending motifs every 10 bp to fix the rotational setting of the DNA as done to position the glucocorticoid hormone-response element (20,21) and TT CPD (22,23). In the T/G motif, (T/A)_3_NN(G/C)_3_, bending is toward the major groove at the central GC base pair of the (G/C)_3_ subunit and toward the minor groove at the central TA base pair of the (T/A)_3_ subunit (24). The sequences were designed so that the sugar phosphate backbone of the ^m^C of the first T= ^m^CG CPD site in the nucleosome (ds1) would be positioned against the histone surface, while the sugar phosphate backbone of the sixth T=^m^C (ds6) would be positioned furthest away from the surface (Figure 2A and B). While there is no guarantee that the CPDs will remain in the same rotational position after being produced by UVB irradiation, hydroxyl radical footprinting showed that T=T CPDs that had been synthetically incorporated into DNA that was phased with TG bending motifs remained in their expected rotational positions (23). In our previous study with this same DNA sequence, hydroxyl radical footprinting showed that the DNA maintained its rotational setting after UVB irradiation, and that a T^m^C CPD designed to be in an outside position remained in its outside position (18). These results indicate that the TG bending motifs are able to maintain the rotational positioning of the DNA in the presence of the multiple CPD and (6-4) photoproducts that are expected to form during irradiation with UVB light (25), though (6-4) photoproducts may be formed 6-fold less frequently in nucleosome DNA than in free DNA (26).

Ten 147-mer DNA duplexes (ds1–10) containing AT^m^CG sites were prepared corresponding to consecutive rotational positions over a full helical turn centered at the dyad axis. Two additional duplexes ds1A and ds6A were also prepared where the 3′-flanking G was replaced by an A to determine the effect of flanking base on the deamination rate at sites positioned against and away from the histone surface (Figure 2A). The duplex DNA substrates were assembled by annealing together independently prepared complementary 147-mer single strands. The 147-mer single strands were prepared by ligating four ODNs together with T4 ligase in the presence of complementary ligation scaffolds (Supplementary Figure S1 and Supplementary Tables S1–S3). Because the method for determining the deamination rates required that the 5′-methylC of the CPD site be 5′-^32^P-labled (13,18), ODN (ts3) was designed to terminate with the ^m^C of the CPD site at the 5′-end so that it could be labeled by polynucleotide kinase prior to ligation. For hydroxyl radical footprinting and Maxam–Gilbert G reaction sequencing, the ODN at the 5′-end of the top strand was 5′-^32^P-end-labeled prior to ligation. The duplex strands were then characterized by gel electrophoresis (Supplementary Figure S2A) and by Maxam–Gilbert G sequencing reaction (Supplementary Figure S3).

### Nucleosome core particle reconstitution with the 147-mer DNA duplexes

To determine optimal conditions for nucleosome core particle preparation, an equimolar mixture of the ten 147-mer DNA duplexes ds1–10 was 5′-end labeled and titrated with the nucleosome core particles (NCPs) isolated from chicken erythrocytes under conditions that promote nucleosome DNA exchange (22). Electrophoresis on a native gel showed that about 95% of 10 nM 147-mer DNA duplexes could be incorporated into NCPs using 1000 nM of the chicken erythrocyte NCPs (Supplementary Figure S2B). The individual 147-mer DNA duplexes ds1–10, ds1A and ds6A were then assembled into nucleosome core particles under these conditions (shown in Supplementary Figure S2C for ds1–10).

### Orientation of the T^m^C sites on the nucleosome core particle

To determine the rotational setting of the DNA on the nucleosome, the equimolar mixture of the ten 5′-end-labeled 147-mer DNA duplexes was subjected to hydroxyl radical footprinting. The hydroxyl radical cleavage pattern exhibited a very pronounced 10–11 bp periodicity, which could be mapped onto the sequence by alignment with the Maxam–Gilbert G reaction bands and with 10 and 25 bp DNA ladders (Figure 2C and Supplementary Figure S3). The ^m^C of ds1, ds2 and ds10, which corresponded to positions 71, 72 and 80 in the sequence, were associated with sites of minimal hydroxyl radical cleavage, indicating that their phosphodiester backbones were against the histone core surface as designed. The ^m^C of ds3 through ds9, which correspond to positions 73–79, were associated with sites of maximal cleavage, indicating that the phosphodiester backbones of these seven ^m^Cs were facing away from the histone core surface.

### Deamination rates of the T=^m^C CPDs

**Figure 3.**
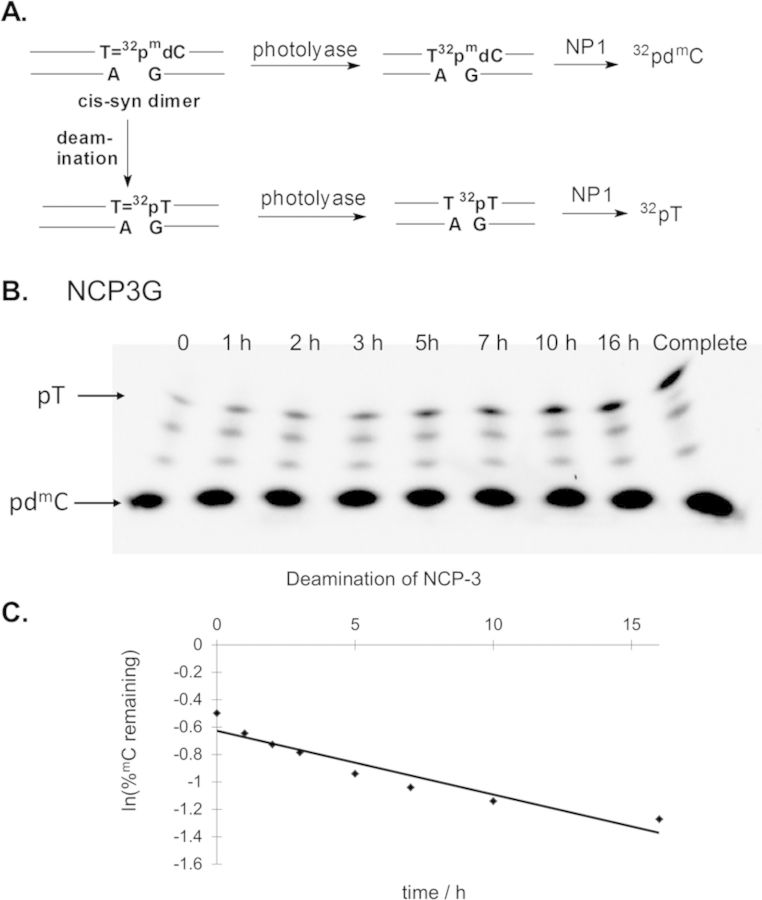
Deamination time course with reconstituted nucleosomes. (A) Scheme for determining the extent of deamination that makes use of site-specifically ^32^P-labeled ^m^dC substrates ds1–10. (B) Chicken blood nucleosomes that had been exchanged with ds3 containing the internally labeled AT^m^CG were irradiated to form AT=^m^CG CPDs that were allowed to deaminate over time, converting ^32^P-^m^C to ^32^P-dT within the CPD. At specific time intervals aliquots of the reaction were removed and photoreverted with photolyase and degraded by P1 nuclease. The samples were first electrophoresed on a 10% denaturing PAGE gel first to separate the digestion products by size. The band containing the ^32^P-^m^dC and ^32^P-dT was then separated by electrophoresis in a pH 3.5 citrate gel as shown in panel (B) with the bands migrating from the bottom to the top. Panel (C) shows the plot of the extent of deamination with time that has been fit to a first order process using an aliquot of each sample that was allowed to deaminate completely to establish the total amount of CPD that was produced.

The deamination rates for the T=^m^CG CPDs were determined by following the conversion of ^32^P-^m^dC to ^32^P-dT by an enzyme-coupled tandem gel electrophoresis assay (Figure 3A) (13,18). In the first step, free or nucleosome-bound 147-mer DNA duplexes were irradiated with 302 nm light to produce the *cis*-*syn* CPDs at the AT^m^CG sites, along with other photoproducts, at 4°C to minimize deamination. The samples were then incubated at 37°C and pH 7.2 to allow deamination to occur and aliquots were removed at various time intervals. An additional aliquot was made to undergo complete deamination for the half-life calculations by lowering the pH to 6.5 and heating the sample at 67°C overnight. The aliquots were then phenol extracted to remove the histone proteins, ethanol precipitated and then incubated with *Escherichia coli* photolyase and visible light to photorevert the *cis*-*syn*-CPDs back to undamaged DNA. The samples were then treated with nuclease P1 to degrade the DNA to mononucleotides. Non-photorevertible dipyrimidine photoproducts, such as (6–4) and Dewar DNA photoproducts, can only be digested to trinucleotides (27) and therefore cannot interfere with the assay. The digested DNA was then electrophoresed on a denaturing gel (TBE) and the mononucleotide-containing band electrophoresed in a second dimension at pH 3.5 (citrate gel) (Figure 3B and Supplementary Figure S4) to separate the deamination product ^32^P-dT from ^32^P-^m^dC, which are then quantified by phosphorimaging.

The rate constants for ^m^C deamination were then determined from the slopes of lines fit to the log of the fraction of remaining T^m^CG CPD (Figure 3C and Supplementary Figure S4) as described in the Experimental Procedures section. The initial amount of T present was non-zero and varied between samples, likely due to varying amounts of deamination that occurred during phenol:chloroform:isopropanol extraction prior to photoreversion with photolyase. Table 1 summarizes the deamination half-lives and yields of the T=^m^CG CPDs in the 10 different rotational positions and in free DNA. When compared with the free DNA, the deamination half-lives of the T=^m^CG CPDs positioned against the histone surface increased by a maximum factor of 3.1, whereas those positioned away decreased by a maximal factor of 3.8. The photoproduct yield for the inside positioned T=^m^CG sites was found to decrease by a maximal factor of 1.7, whereas the outside positioned sites increased by a maximal factor of 1.9. The observation of a rotational dependence on the deamination rates indicates that the CPDs were being largely maintained in a fixed relative rotational position to each other in spite of the likely presence of other photoproducts within the DNA. A fixed rotational setting is also indicated by the similar deamination rates of the CPD in position 1 and the CPD at position 10 in spite of being nine nucleotides apart, as would be expected if they were in nearly the same rotational position.

**Table 1.** Nucleosome rotational positioning effect on T=^m^CG CPD and T=^m^CA photoproduct yield and deamination half life

	T = ^m^C yield (%)	Fold change	Deamination half-life (h)	Fold change
Free ds1-10	12	-	13 ± 1	-
NCP ds1	6.9	0.6	41 ± 2	3.1
NCP ds2	7.6	0.6	39 ± 3	3.0
NCP ds3	13	1.1	15 ± 2	1.2
NCP ds4	14	1.2	9 ± 1	0.7
NCP ds5	19	1.6	5 ± 1	0.4
NCP ds6	22	1.9	3.5 ± 0.3	0.3
NCP ds7	20	1.7	3.6 ± 0.3	0.3
NCP ds8	14	1.2	10 ± 0.5	0.8
NCP ds9	12	1.0	12 ± 0.5	0.9
NCP ds10	8.4	0.7	36 ± 2	2.8
Free ds1A	14	-	85 ± 6	-
NCP ds1A	12	0.8	117 ± 7	1.3
Free ds16A	14	-	90 ± 3	-
NCP ds6A	24	1.7	28 ± 0.6	0.32

The two T=^m^CA CPDs deaminated with significantly different kinetics than the corresponding T=^m^CG CPDs (Table 1, Supplementary Figure S5). While the most outward positioned AT= ^m^CA DNA photoproduct deaminated 3.1-fold faster than in free DNA, the inside positioned AT=^m^CA, unlike the corresponding AT=^m^CG DNA photoproduct, did not show any significant decrease in deamination rate, compared to free DNA. Compared with the 12-fold difference in deamination rate between inward and outward positioned T=^m^CG CPDs, the T=^m^CA CPDs only exhibited a 4-fold difference in deamination rate.

## DISCUSSION

**Figure 4.**
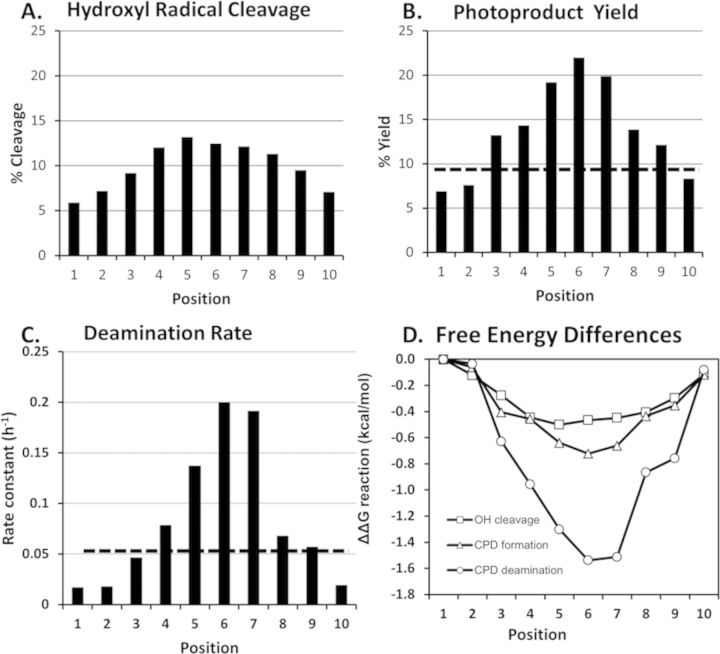
Photoproduct yield and deamination half-lives as a function of rotational position. (A) Bar graph showing the relative percent of hydroxyl radical cleavage at each rotation position of the ^m^C in the nucleosome core particle. (B) Bar graph of T^m^C CPD yield at each rotational position compared to that in free DNA (dashed line). (C) Bar graph of the deamination rate constants for the T^m^C CPDs at each rotational position compared to that in free DNA (dashed line). (D) Relative differences between the transition state free energies for hydroxyl radical cleavage, photoproduct formation and deamination at each rotational position.

In our previous study, we examined photoproduct formation and deamination at two rotational positions, one where the sugar phosphate backbone of ^m^C in T^m^CG was furthest from the histone surface (corresponding to position 6) and a second that was closest to the surface (corresponding to position 1) (Figure 2B). At that point, we did not know whether or not these positions corresponded to the extremes of the deamination rates. In the current study, however, we found that the deamination rates varied over a 12-fold range and were maximal and minimal at these same two positions (Figure 2B). These outward and inward facing positions also coincide with the maximal and minimal rates of photoproduct formation (positions 6 and 1, Figure 4B) and hydroxyl radical cleavage (positions 5 and 1, Figure 4C), as generally reported by others (3,4,24,28). It is not obvious, however, why the maximal and minimal rates for all three types of reactions should coincide with the same rotational positions.

**Figure 5.**
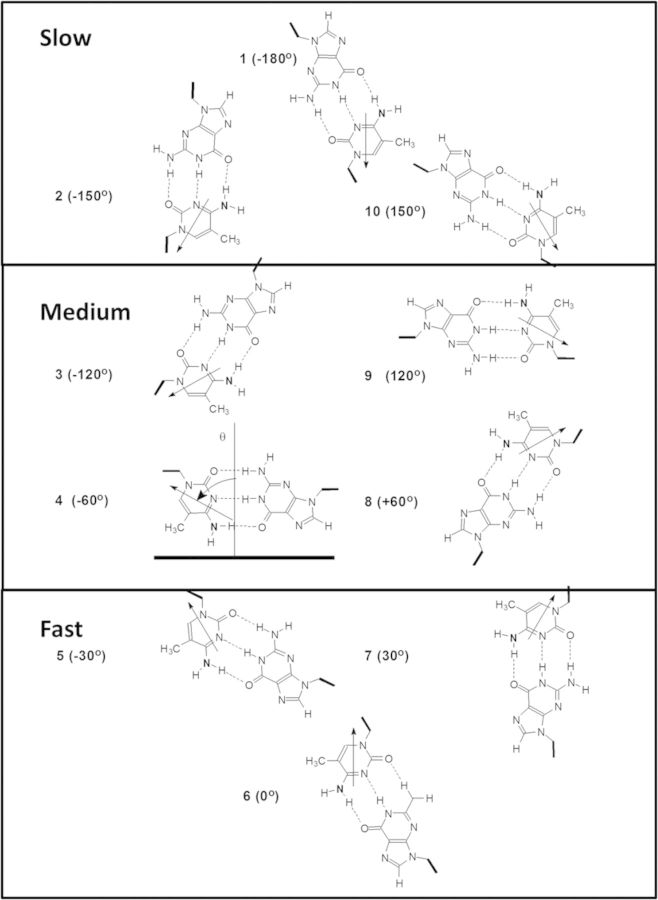
Orientations of the G•^m^C base pairs relative to the histone surface. Orientations were approximated from the crystal structure of a nucleosome core particle 1KX5.pdb and arranged from top to bottom in order of slowest to fastest photoproduct formation and deamination. The rates fit best as a function of the angle between a vector bisecting the C6-N1 and N3-C4 bonds and a perpendicular vector to the histone core surface.

First, consider the chemistry for hydroxyl radical cleavage and photoproduct formation. Hydroxyl radical cleavage of DNA with iron•EDTA results mainly from initial abstraction of the 5′,5″ hydrogens from the sugar backbone (29). The most exposed hydrogens are those facing away from the histone surface, and the least exposed are those facing toward the histone surface (Figures 2B and 5), which agree with the observed maxima and minima at 5 and 1. In contrast, CPD formation involves a photochemical cycloaddition reaction between the 5,6-double bonds of adjacent pyrimidines. It had been previously observed that CPD formation was maximal about 1.5 nucleotides to the 3′ of the dyad axis of the nucleosome. Because DNA bends toward the major groove at the dyad axis and because molecular mechanics calculations (30), which were later supported by crystal structure data, indicated that CPDs also bend DNA toward the major groove (10), it was proposed that DNA bending controlled photoproduct formation (4–6). If so, CPD formation should be maximal at position 4 and not at position 6 as observed in our study (Figure 5). The lack of exact correspondence between the predicted and observed site of maximal CPD formation is consistent with the Hammond postulate (31) for exothermic reactions, such as photodimerization, which postulates that the transition state will more closely resemble the starting conformation than the product conformation.

A recent experimental study found that CPD formation occurs within a picosecond of photoexcitation, which requires that the double bonds are properly oriented at the time of excitation (32). Subsequent molecular dynamics calculations have shown that the quantum yield depends on the fraction of conformations that have the two reacting double bonds within a given distance and angle of each other (33,34). This would suggest that the outer positions either are in a more favorable conformation than the inner positions, or that they have a greater fraction of photoreactive conformations than the inner positions. Replacement of the base pairs corresponding to positions 1–10 in the high resolution (1.9 Å) crystal structure of a 147-mer nucleosome core particle 1KX5.pdb (35) with TA base pairs did not, however, show any strong correlation between interbond distance and improper torsion angle with photoproduct yield except for position 10 (Supplementary Figure S6).

**Figure 6.**
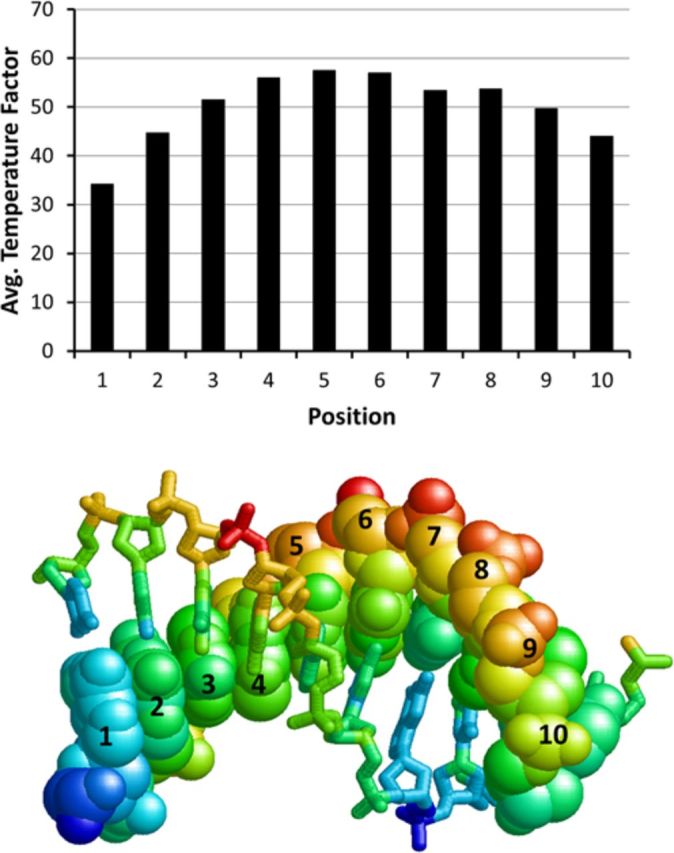
Temperature factors for a high resolution nucleosome core particle structure. (A) Plot of average temperature factors for all atoms of the nucleotide in the 1KX5.pbd structure that corresponds to the indicated position of the ^m^C. (B) Structure of the −3 to +6 section of the 1KX5.pdb structure shown in spacefill corresponding to the ^m^C sites numbered 1–10 in this study, colored according to their relative temperature factors with red as the highest and blue as the lowest. The complementary strand, −6 to +3, is shown in wireframe and colored in the same way as for the ^m^C containing strand.

It was also proposed that DNA flexibility explains the variation of CPD formation with rotation position since the DNA in contact with the histone surface would be less mobile than the DNA at outside positions (3). The difference in flexibility at inside and outside positions can be assessed from temperature factors taken from the high resolution 1KX5.pdb structure (35) (Figure 6). The maximum average temperature factor occurs at position 5 and the minimum at position 0. For another high resolution nucleosome structure (3UT9.pdb), the maximum and minimum were at 6 and 1 (36) (Supplementary Figure S7). Since CPDs also form preferentially at outside positions in protein-free DNA loops, it appears that DNA curvature relating to flexibility and motion, rather than direct protein-DNA contacts, may be controlling DNA photoreactivity (5). Since outside loop positions are less constrained (more flexible) than the inside positions, it is reasonable that a greater fraction of the conformations at outside positions will favor photodimer formation than those inside, resulting in a maximum yield at position 6 and minimum at position 1. This explains why photoproduct yield depends on the orientation of the ^m^C relative to the most flexible position, rather than on the orientation of the major groove. The photoproduct formation rates appear to track best with the relative angle between a vector bisecting the N3-C4 and N1-C6 bonds of the methyl C and a perpendicular vector from the histone surface (Figure 5).

Deamination of C in a *cis*-*syn* CPD is more complicated than hydrogen abstraction or photodimerization, and proceeds through either attack of water or hydroxide on the C4 carbon of cytosine that is protonated at N3 (37) to give a tetrahedral intermediate (Figure 1A). This suggests that deamination would be fastest when the C4 position is most exposed as for hydroxyl radical cleavage. Since the C4 carbonyl lies in the major groove of the DNA, it would be maximally exposed when facing away from the histone surface at position 9, and not at 6, which shows the fastest deamination rate (Figure 5). Likewise, the major groove is minimally exposed when it is facing the histone surface which corresponds to position 4, and not at 1, which shows the slowest deamination rate. Furthermore, as we had noted in our previous study, the C4 positions of the ^m^Cs at positions 1 and 6 (Figure 5) are equally unobstructed by protein within a radius of 8.5 Å (1KX5.pdb), yet have drastically different rates of deamination (18). Thus, it appears that water accessibility is not the major factor controlling deamination in nucleosomes. On the other hand, DNA flexibility might explain the differences in deamination rates. The more flexible outside positions may facilitate base pair opening and N3 protonation and/or subsequent water attack on the C4 position of the ^m^C of a CPD. A CPD at an inside position, however, might be much more restricted in its movements due to the compressed and constrained nature of the phosphodiester backbone. Similar to CPD formation, DNA flexibility could explain why the deamination rate also tracks with the position of the ^m^C relative to the most outward position, rather than with the orientation of the major groove.

One type of motion that might increase deamination in nucleosomes is a CPD flip-out mechanism similar to that catalyzed by CPD photolyases (38,39). This type of motion would expose the N3 of the CPD for protonation a necessary step in the deamination reaction (37), and could conceivably be enhanced by DNA bending. The two crystal structures for both Type I and Type II photolyases show that the DNA becomes bent upon binding, and that the CPD is flipped out at the outer part of the bend, where the deamination rate is found to be maximal in the nucleosome core particle. If nucleosomes facilitate CPD ‘flip out’ in this manner, one might expect a CPD in an outer position to show enhanced repair by DNA photolyase. A study of CPDs in T-tracts in a rotationally phased nuclesosome core particle found, however, that repair by photolyase is greatly inhibited relative to free DNA and that sites of more efficient repair do not correlate with their rotational position (40) indicating that steric and other factors may be more important in CPD recognition by photolyase. Another possibility is that a base opposite the CPD is flipping out as observed for T4 endonuclease V binding to a CPD (41), or as deduced from a recent proton exchange nuclear magnetic resonance study on base pair opening in a CPD-containing DNA duplex (42). This possibility is less likely, however, since the bases opposite a CPD in position 6 which has the fastest deamination rate, have the lowest temperature factors and would be predicted to have the least flexibility since they are held against the histone core surface.

The effect of rotational position is much greater on deamination than either hydroxyl radical cleavage or photoproduct formation as seen from the plot of ΔΔG^‡^ versus rotational position (Figure 4D). Whereas ΔΔG^‡^ amounts to only about 0.4–0.6 kcal/mol between outermost and innermost positions for hydroxyl radical cleavage and photoproduct formation, it is about three times larger for deamination, or about 1.5 kcal/mole. This greater effect may be due in part to a significant flanking base effect on deamination rate. It was previously discovered that a 3′-flanking G greatly accelerates the deamination of ^m^C in a T = ^m^CG CPD in duplex DNA compared to a 3′-flanking A, and that the O6 carbonyl of the G was the functional group most responsible for this difference (13). In that study, AT^m^CG deaminated 25-fold faster than AT^m^CA (*t*_1/2_'s of 7 and 175 h, respectively) while in this study AT^m^CG deaminated only 7-fold faster than AT^m^CA (*t* $\frac{1}{2}$'s of 13 and 85 h). When in the outermost nucleosome position 6, the deamination rate of both T^m^CG and T^m^CA CPDs increased to a similar extent relative to free DNA (3.7-fold versus 3.2-fold), but when in the innermost position the rate decreased by 3.1-fold for T^m^CG but only 1.3-fold for T^m^CA (Table 1). This suggests that the G at the innermost position is held in a less favorable position for catalyzing the deamination than in free DNA, while the effect is much less for A which is unable to catalyze the deamination. Some of this effect may also be due to the fact that the DNA at the dyad is unwound relative to free DNA (28).

The difference in deamination rates between the innermost and outermost CPDs were much greater in our previous study (45-fold versus 12-fold) (18). Most of this difference can be attributed to the outside position which deaminated 9-fold faster than in free DNA in the previous nucleosome construct compared to 3.1-fold faster observed in the current construct. The inside position showed less difference, deaminating 4.7-fold slower in the previous sequence compared to 3.5-fold slower in this sequence. The slightly different sequence contexts of the two CPDs used in the previous construct, GT^m^CG inside and AT^m^CG outside, is unlikely to explain the difference, since their deamination half-lives were similar in free DNA (12.2 ± 0.9 h versus 14.0 ±1.3 h) and were essentially the same as we observed for the current construct (13 ± 1 h). A more likely explanation is that the sequence used in the previous nucleosome construct was based on a sequence used to rotationally position two TT CPDs for DNA repair studies (23) that had been modified from a properly phased sequence by the insertion of three extra nucleotides near the dyad axis (22). The addition of the three nucleotides would have caused the G/T positioning sequences at the 3′-side of the 150-mer to be about 104° out of phase with those at the 5′-side. This phase difference could have affected the structure and dynamics of the DNA, possibly causing local unwinding or kinking at the dyad axis or equilibration between different translational positions, which might have affected the deamination rates. It is not clear, however, why it would have enhanced the deamination rates of the outside CPDs more than the inside ones. Whatever the effect was, it did not seem to have as dramatic an effect on the photoproduct yield, so the origin of the difference in deamination rates between the two sequences remains to be elucidated.

When averaged for all 10 rotational positions, we calculate that the average deamination rate constant for a T=^m^CG CPD is 1.6-fold higher than in free DNA (0.083 h^−1^ versus 0.053 h^−1^). This result is quite different than what has been deduced for the spontaneous deamination of undamaged C deduced from a global analysis of C to T mutations in yeast, which concluded that nucleosomes completely suppress deamination (43). While both deamination mechanisms require protonation and attack of water or hydroxide on C4, CPDs distort DNA structure and may be more susceptible to base pair opening or CPD flipping by the nucleosome than a normal base pair.

**Figure 7.**
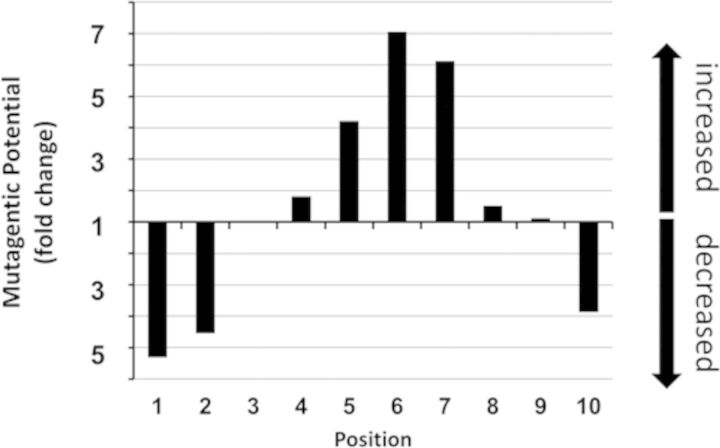
Mutagenic potential of T^m^CG sites as a function of rotational position. The product of the relative rate of CPD formation and CPD deamination is plotted versus rotational position.

Given that sunlight-induced C to T mutations arise principally from the deamination bypass mechanism, the mutagenic potential of a T^m^CG site as a function of rotational position is expected to depend on the rate of photoproduct formation, deamination, repair and replication. The mutation potential is expected to increase with an increase in the rate of photoproduct formation and deamination, and with lower rates of repair and replication which would allow more time for deamination. We have shown that the rates of photoproduct formation and deamination are affected by rotational position in the same way and thereby reinforce each other. The mutagenic potential as a function of rotational position would therefore correspond to the product of these rates, and greatly increases for T^m^CG sites positioned away from the surface and greatly decreases for those positioned against the surface compared to free DNA (Figure 7). At position 6 the mutagenic potential is 7-fold higher than free DNA, and at position 1 it is 5.3-fold lower.

The effect of transcription-coupled repair and replication on the mutagenic potential of a T^m^CG site, however, is expected to be independent of rotational position, since nucleosomes would be absent when the polymerases encounter the CPD. The overall mutagenic potential, however, is expected to be lower in the transcribed strand of actively transcribed genes than in non-transcribed genes. It is unclear, however, whether global genome nucleotide excision repair is sensitive to the rotational position of a CPD. While an early *in vivo* study indicated that there was no pronounced selectivity for rotational position (44), a more recent *in vitro* study found that an outside facing CPD at the dyad was repaired only 1.5-fold faster than one facing inside (23). Nucleosomes, however, have been found to greatly suppress global genome repair *in vivo* by factors of 2–10 (23), which would greatly increase the relative mutagenic potential of more compact chromatin compared to more open chromatin.A recent analysis of translational positioning of nucleosomes in the human genome indicates that only about 0.3% are strongly positioned, 8.4% are moderately strongly positioned and 84% are weakly positioned (45). Though the translational positioning may be weak, there was evidence that the rotational positioning is maintained, which would maintain the mutagenic potential of a particular site over multiple nucleosome arrangements. In the case of weakly rotationally positioned nucleosomes, one might expect to see an averaged mutagenic potential, which is calculated to be 2.3-fold greater than for free DNA for the 10 sites studied, if one ignores the effects of replication and repair. Alternatively, it may be that after formation, a CPD will cause repositioning of the nucleosome so that the CPD can adopt an outside position which favors bending of the CPD toward the major groove (9). If it were to equilibrate to position 4, the deamination rate would only be 1.2-fold higher than in free DNA and the overall mutagenic potential would depend on the average position it was in during photoproduct formation.

## CONCLUSION

We have determined that the rotational position of a T^m^CG site in a full turn of nucleosome at the dyad axis greatly affects the rate of CPD formation and deamination in a synergistic manner. The combined effect results in a large modulation of the potential for a C to T mutation arising from a deamination bypass mechanism which could therefore help explain in part the origin of UV mutation hotspots and coldspots. It remains to be seen how the rate of CPD formation and deamination is modulated by rotational position at other translational positions within the nucleosome, where differences in DNA structure and the presence of histone tails may have an effect, as has been discovered for the reactivity of abasic sites (46). In addition, it will be important to determine the effect the H1 linker protein which interacts with the nucleosome at the dyad axis as well as the incoming and outgoing DNA in higher forms of chromatin (47) on photoproduct formation and deamination. Based on our results with CPDs, we also expect nucleosome structure to similarly modulate the UV mutagenic potential of C^m^CG sites and unmethylated TC, CT and CC sites, as well as other types of damage that also undergo deamination, such as 5,6-dihydroxy-5,6-dihydrocytosine (48,49). It would also be interesting to know whether the human genome has evolved to minimize the mutagenicity of UV light by selecting against T^m^CG sites in exons at outside positions in strongly positioned nucleosomes, and will require further study. An evolutionary response to weakly positioned nucleosomes, such as in the p53 tumor suppressor gene, (50) may explain why there is an absence of T^m^CG sites in the non-transcribed strand of this gene in humans but not in mice (16).

## SUPPLEMENTARY DATA

Supplementary Data are available at NAR Online.

SUPPLEMENTARY DATA
