# Supplementary material for: Synergistic modulation of cyclobutane pyrimidine dimer photoproduct formation and deamination at a TmCG site over a full helical DNA turn in a nucleosome core particle
Source: Nucleic Acids Res. 2014 Nov 11;42(21):13122–33. doi: 10.1093/nar/gku1049 (PMC4245940; doi:10.1093/nar/gku1049)
Supplement: SUPPLEMENTARY DATA [file supp_42_21_13122__index.html]

Synergistic modulation of cyclobutane pyrimidine dimer photoproduct formation and deamination at a TmCG site over a full helical DNA turn in a nucleosome core particle — Synergistic modulation of cyclobutane pyrimidine dimer photoproduct formation and deamination at a TmCG site over a full helical DNA turn in a nucleosome core particle — SUPPLEMENTARY DATA 

# Synergistic modulation of cyclobutane pyrimidine dimer photoproduct formation and deamination at a TmCG site over a full helical DNA turn in a nucleosome core particle

## SUPPLEMENTARY DATA

**Files in this Data Supplement:**

- SUPPLEMENTARY DATA
