# Supplementary material for: Synergistic modulation of cyclobutane pyrimidine dimer photoproduct formation and deamination at a TmCG site over a full helical DNA turn in a nucleosome core particle
Source: Nucleic Acids Res. 2014 Nov 11;42(21):13122–33. doi: 10.1093/nar/gku1049 (PMC4245940; doi:10.1093/nar/gku1049)
Supplement: SUPPLEMENTARY DATA [file supp_gku1049_nar-02004-d-2014-File009.pdf]

Supplementary Information for:

Synergistic Modulation of Cyclobutane Pyrimidine Dimer Photoproduct Formation and Deamination at a T^{m}CG Site Over a Full Helical DNA Turn in a Nucleosome Core Particle

Qian Song[1], Vincent J. Cannistraro[1], and John-Stephen Taylor[1,*]

Department of Chemistry, Washington University, St. Louis, MO 63130

*To whom correspondence should be addressed. Tel: +1 314 935 6721; Fax: +1 314 935 4481;

Email: taylor@wustl.edu

Figure	Page
Table S1. Common ODN sequences for all ligations:	S3
Table S2. ODNs for 147-mer ds1-10	S4
Table S3. ODNs for 147-mer ds6A	S7
Figure S1. Ligation scheme for preparing 147-mers containing rotation-specific T^{m}CG sites.	S8
Figure S2. Reconstitution of the nucleosome core particles with the 147-mer DNA duplexes.	S9
Figure S3. Hydroxyl radical footprinting of the nucleosome core particles and DMS sequencing of the DNA 147-mer duplexes.	S10
Figure S4. Deamination rates of T=^{m}C CPD in nucleosome-bound ds1-10 and free ds1-10 combined.	S11-12
Figure S5. Deamination rate of T=^{m}CA CPD in nucleosome-bound and nucleosome-free ds1A & 6A .	S14
Figure S6. Relative orientation between adjacent C5-C6 bonds in T-tracts derived from crystal structures of nucleosome core particles.	S15
Figure S7. Temperature factors for the nucleotides in 3UT9.pbd corresponding to the ^{m}C positions in the current study.	S16

Table S1. Common ODN sequences for all ligations:

Top strand (ts):

ODN	Sequence
ts1	AGT TAT GTT AGA GCC TGT AAC TCG GTG TTA GAG CCT
ts4	CGG TGT TAG AGC CTG TAA CTC GGT GTT AGA GCC TG

Top strand ligation scaffolds (tls)

ODN	Sequence
tls12	ACC GAG TTA CAG GCT CTA AC
tls34	TCT AAC ACC GAG TTA CAG GC

Bottom strand (bs)

ODN	Sequence
bs1	CAG GCT CTA ACA CCG AGT TAC AGG CTC TAA CAC CGA GTT
bs3	CAC CGA GTT ACA GGC TCT AAC ACC GAG TT
bs4	ACA GGC TCT AAC ACC GAG TTA CAG GCT CTA ACA TAA CT

Bottom strand ligation scaffolds (bls)

ODN	Sequence
bls12/bls34	TAGAGCCTGTAACTCGGTGT

Table S2. Specific ODNs for 147-mer ds1-10

ODN	Sequence
ts2_1	GTA ACT CGG TGT TAG AGC CTG TAA CTC GGT GTA T
ts3_1	^{m}CG ATG GTA TAG AGC CTG TAA CAG AAT GTT AGA GCC TGT AAC T
tls23_1	TAT ACC ATC GAT ACA CCG AG
bs2_1	ACA GGC TCT AAC ATT CTG TTA CAG GCT CTA TAC CAT CGA TA
bls23_1	TAA CTC GGT GTA TCG ATG GT
ts2_2	GTA ACT CGG TGT TAG AGC CTG TAA CTC GGT GAT AT
ts3_2	^{m}CG ATA TGT ATA GCC TGT AAC AGA ATG TTA GAG CCT GTA ACT
tls23_2	ATA CAT ATC GAT TAC ACC GA
bs2_2	ACA GGC TCT AAC ATT CTG TTA CAG GCT ATA CAT ATC GAT AT
bls23_2	TAA CTC GGT GAT ATC GAT TA
ts2_3	GTA ACT CGG TGT TAG AGC CTG TAA CTC GGT GTA TAT
ts3_3	^{m}CG ATA TGT AAG CCT GTA ACA GCC TGT TAG AGC CTG TAA CT
tls23_3	TTA CAT ATC GAT ATA CAC CG
bs2_3	ACA GGC TCT AAC AGG CTG TTA CAG GCT TAC ATA TCG ATA TA
bls23_3	TAA CTC GGT GTA TAT CGA TA
ts2_4	GTA ACT CGG TGT TAG AGC CTG TAA CTC GGT GAT ACA T
ts3_4	^{m}CG TGT ATG AGC CTG TAA CAG CCT GTT AGA GCC TGT AAC T
tls23_4	CAC ATA CAC GAT GTA TCA CC
bs2_4	ACA GGC TCT AAC AGG CTG TTA CAG GCT CAT ACA CGA TGT AT
bls23_4	TAA CTC GGT GAT ACA TCG TG
ts2_5	GTA ACT CGG TGT TAG AGC CTG TAA CTC GGT GTA TGT AT
ts3_5	^{m}CG ATA GTA GCC TGT AAC AGC CTG TTA GAG CCT GTA ACT
tls23_5	GCT ACT ATC GAT ACA TAC AC
bs2_5	ACA GGC TCT AAC AGG CTG TTA CAG GCT ACT ATC GAT ACA TA
bls23_5	TAA CTC GGT GTA TGT ATC GA

ts2_6	GTA ACT CGG TGT TAG AGC CTG TAA CTC GGT GAT GAT GAT
ts3_6	^{m}**C**G TAT GAG CCT GTA ACA GCC TGT TAG AGC CTG TAA CT
tls23_6	GGC TCA TAC GAT CAT CAT CA
bs2_6	ACA GGC TCT AAC AGG CTG TTA CAG GCT CAT ACG ATC ATC AT
bls23_6	TAA CTC GGT GAT GAT GAT CG
ts2_7	GTA ACT CGG TGT TAG AGC CTG TAA CTC GGT GTA TAT GCA T
ts3_7	^{m}**C**G TAC AGC CTG TAA CAG CCT GTT AGA GCC TGT AAC T
tls23_7	AGG CTG TAC GAT GCA TAT AC
bs2_7	ACA GGC TCT AAC AGG CTG TTA CAG GCT GTA CGA TGC ATA TA
bls23_7	TAA CTC GGT GTA TAT GCA TC
ts2_8	GTA ACT CGG TGT TAG AGC CTG TAA CTC GGT GAT ACG CACA T
ts3_8	^{m}**C**G TAA GCC TGT AAC AGC CTG TTA GAG CCT GTA ACT
tls23_8	CAG GCT TAC GAT GTG CGT AT
bs2_8	ACA GGC TCT AAC AGG CTG TTA CAG GCT TAC GAT GTG CGT AT
bls23_8	TAA CTC GGT GAT ACG CAC AT
ts2_9	GTA ACT CGG TGT TAG AGC CTG TAA CTC GGT GTA TGT AGA CAT
ts3_9	^{m}**C**G CAG CCT GTA ACA GCC TGT TAG AGC CTG TAA CT
tls23_9	ACA GGC TGC GAT GTC TAC AT
bs2_9	ACA GGC TCT AAC AGG CTG TTA CAG GCT GCG ATG TCT ACA TA
bls23_9	TAA CTC GGT GTA TGT AGA CA
ts2_10	GTA ACT CGG TGT TAG AGC CTG TAA CTC GGT GAT ACG CAC GTA T
ts3_10	^{m}**C**G AGC CTG TAA CAG CCT GTT AGA GCC TGT AAC T
tls23_10	TAC AGG CTC GAT ACG TGC GT
bs2_10	ACA GGC TCT AAC AGG CTG TTA CAG GCT CGA TAC GTG CGT AT
bls23_10	TAA CTC GGT GAT ACG CAC GT

Table S3. ODNs for 147-mer ds-1A

ODN	Sequence
ts2_1A	GTA ACT CGG TGT TAG AGC CTG TAA CTC GGT GTA T
ts3_1A	^{m}**C**A ATG GTA TAG AGC CTG TAA CAG AAT GTT AGA GCC TGT AAC T
tls23_1A	TAT ACC ATT GAT ACA CCG AG
bs2_1A	ACA GGC TCT AAC ATT CTG TTA CAG GCT CTA TAC CAT TGA TA
bls23_1A	TAA CTC GGT GTA TCA ATG GT
ts2_6A	GTA ACT CGG TGT TAG AGC CTG TAA CTC GGT GAT GAT GAT
ts3_6A	^{m}**C**A TAT GAG CCT GTA ACA GCC TGT TAG AGC CTG TAA CT
ts23_6A	GGC TCA TAT GAT CAT CAT CA
bs2_6	ACA GGC TCT AAC AGG CTG TTA CAG GCT CAT ATG ATC ATC AT
bls23_6	TAA CTC GGT GAT GAT GAT CA

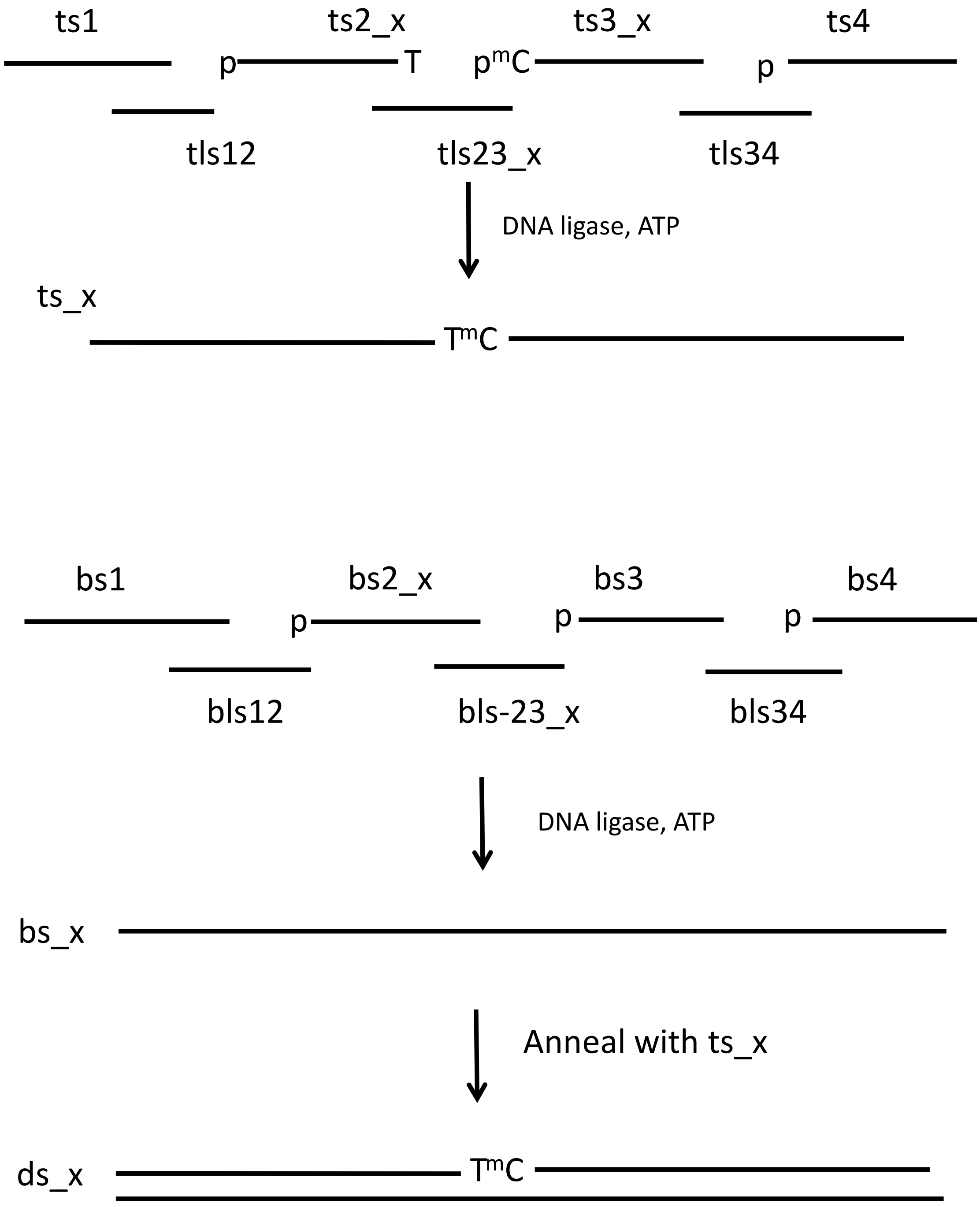

Figure S1. Ligation scheme for preparing 147-mers containing rotation-specific T^{m}CG sites. The ^{m}C of the CPD site could be specifically ^{32}P-radiolabeled by treatment of unphosphorylated ODN ts3_x with γ-^{32}P-ATP and kinase.

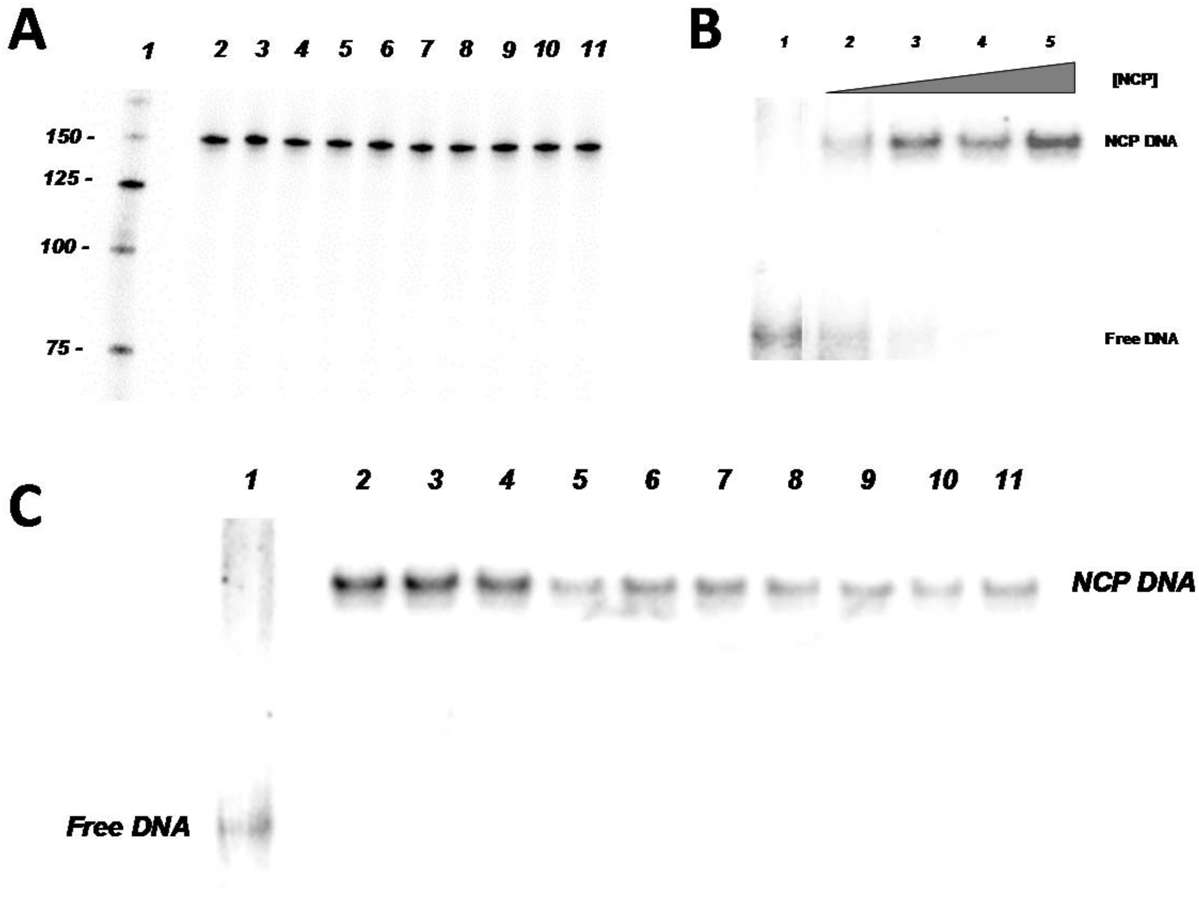

Figure S2. Reconstitution of the nucleosome core particles with the 147-mer DNA duplexes. A) The PAGE-purified single strand 147-mers were annealed together to form duplexes and characterized by native gel electrophoresis on a 10% acrylamide, 0.3% bisacrylamide polyacrylamide gel in TBE. Lane 1: 25 bp DNA ladder, lane 2-11: 147-mer ds1-10. Each duplex substrate was isolated from the gel for further studies B) An equimolar mixture of the ten 147-mer DNA duplexes ds1-10 (10 nM duplex DNA) was incubated with increasing amounts of chicken erythrocyte nucleosome core particle (NCP) (lanes 1-4: 100, 300, 500, 1000 nM) at room temperature in 2 M NaCl at pH 7.5 for 2 h followed by dialysis overnight at 4°C in 50 mM NaCl, with final equilibration at 55°C for 2 h. The reconstituted NCP were then electrophoresed on a native polyacrylamide gel (6% acrylamide, 0.2% bisacrylamide in TBE). C) Individual 147-mer DNA duplexes ds1-10 (10 nM each) were incubated with 1000 nM chicken erythrocyte nucleosome core particles (NCP) at room temperature in 2 M NaCl at pH 7.5 for 2 h followed by dialysis overnight at 4°C in 50 mM NaCl, with final equilibration at 55°C for 2 h. The reconstituted NCP were then electrophoresed on a native polyacrylamide gel (6% acrylamide, 0.2% bisacrylamide in TBE). Lane 1: free 147-mer DNA duplex, Lane 2-11: reconstituted nucleosome-bound ds1-10.

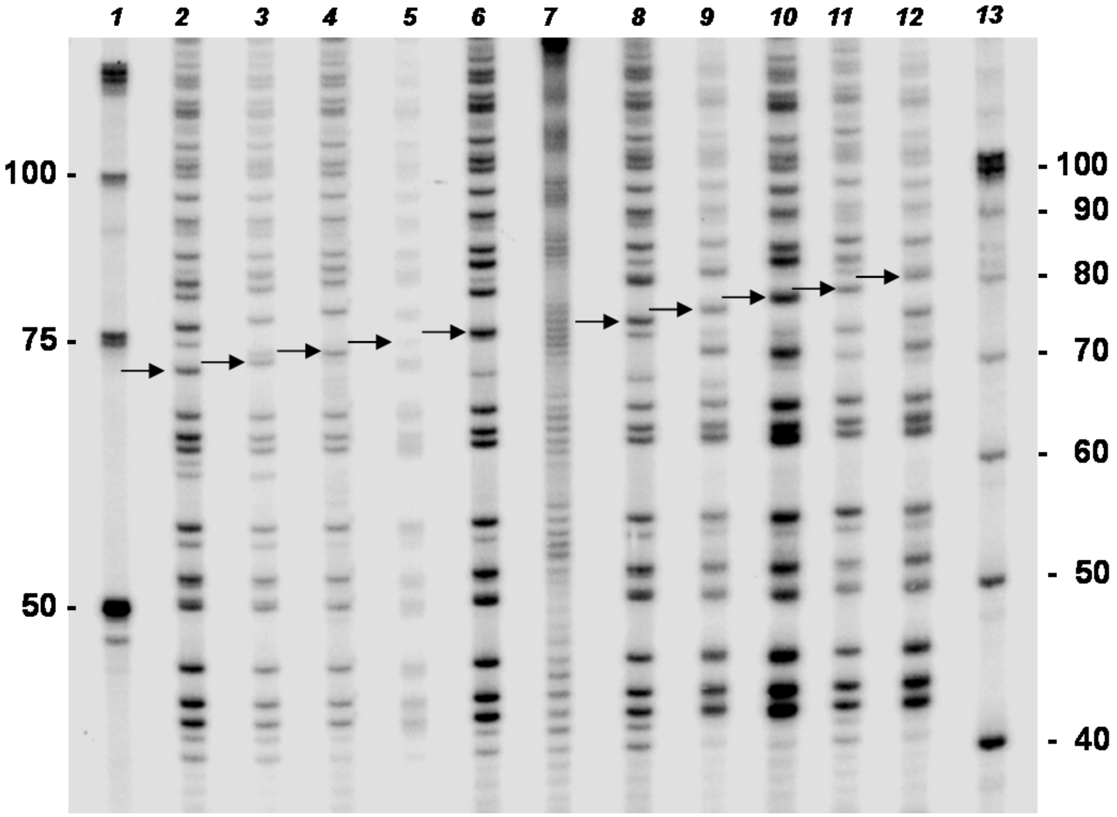

Figure S3. Hydroxyl radical footprinting of the nucleosome core particles and DMS sequencing of the DNA 147-mer duplexes. The nucleosome core particle was reconstituted with an equimolar mixture of 5'-end-labeled 147-mer DNA duplexes ds1-10 and then subjected to hydroxyl radical footprinting and electrophoresis on a 7 M urea, 10% acrylamide, 0.3% bisacrylamide denaturing gel. Lane 1, 25 bp DNA ladder; lane 2 to 6, Maxam Gilbert G reaction on ds1-5; lane 7, hydroxyl radical footprinting of the reconstituted nucleosome core particle with the equimolar mixture of the ten 147-mer DNA duplexes ds1-10; lane 8 to 12, Maxam Gilbert G reaction on ds6-10; lane 13, 10 bp DNA ladder. Arrows point to the G of the T^{m}CG sites in ds1-10.

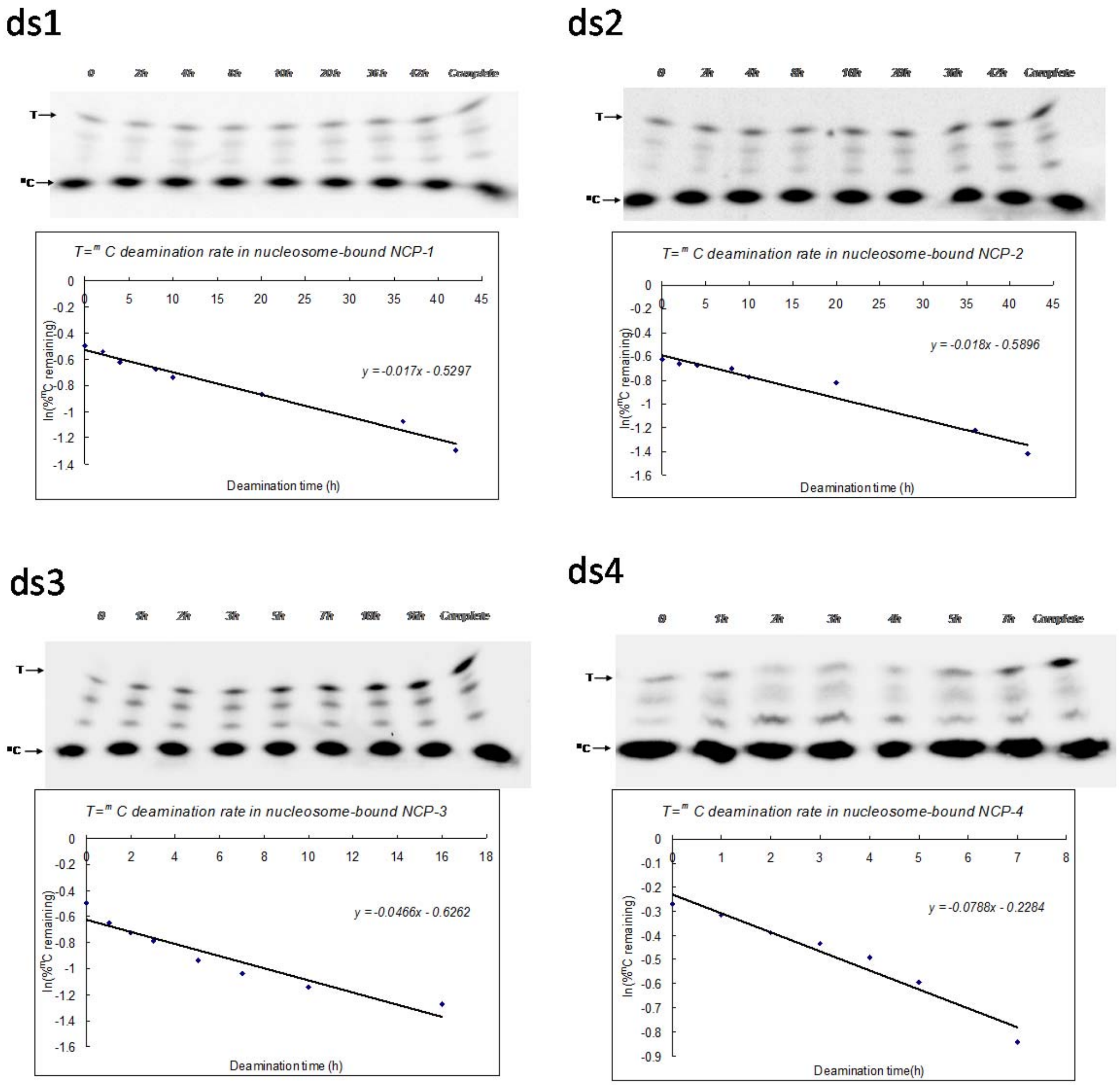

Figure S4. Deamination rates of T=^{m}C CPD in nucleosome-bound ds1-10 and free ds1-10 combined. Gel electrophoresis of the NP degradation products of the photoreverted DNA as a function of deamination time. Plots of the fraction ^{m}C remaining in the T^{m}C CPD vs time.

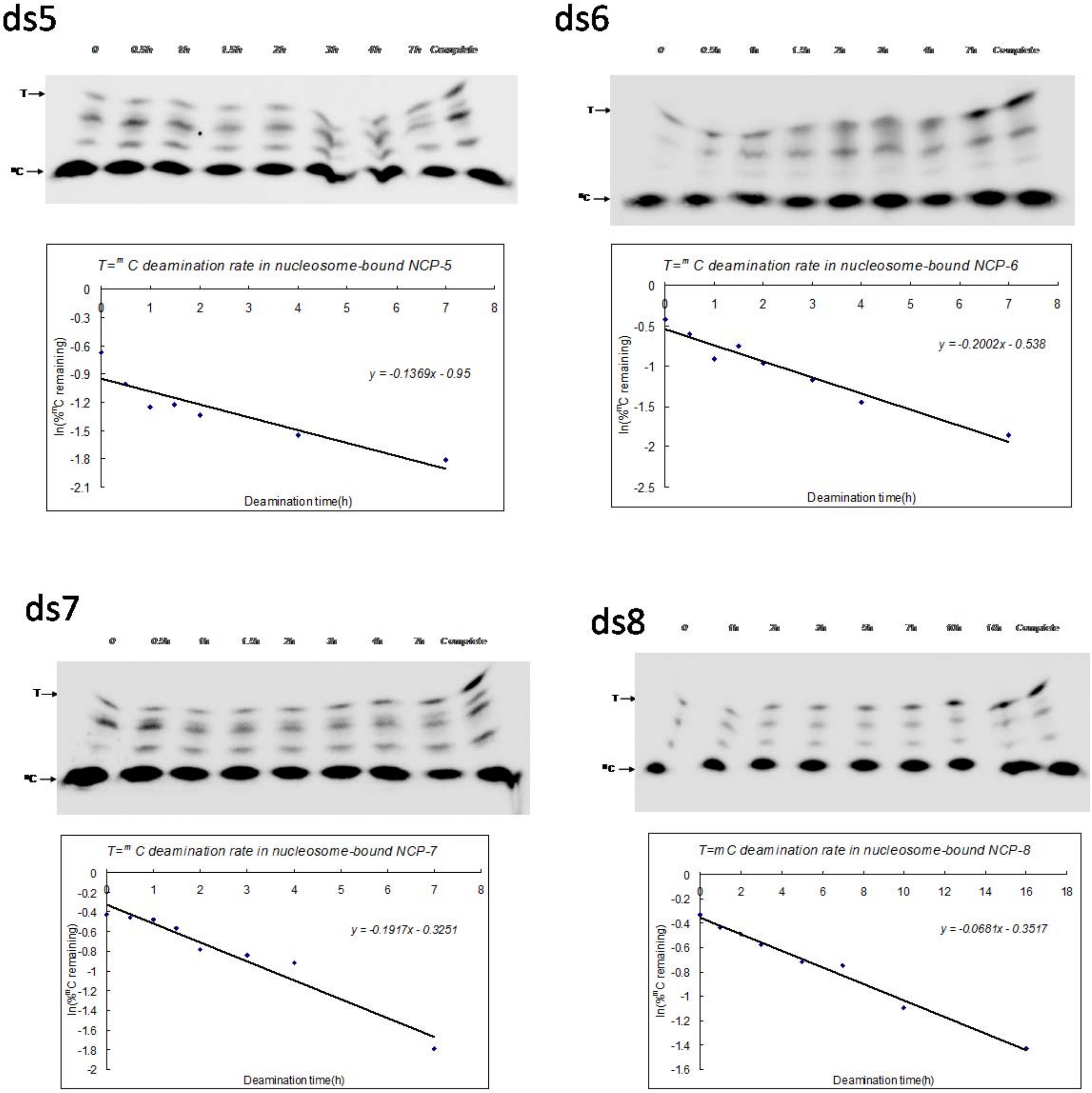

Figure S4 (cont.)

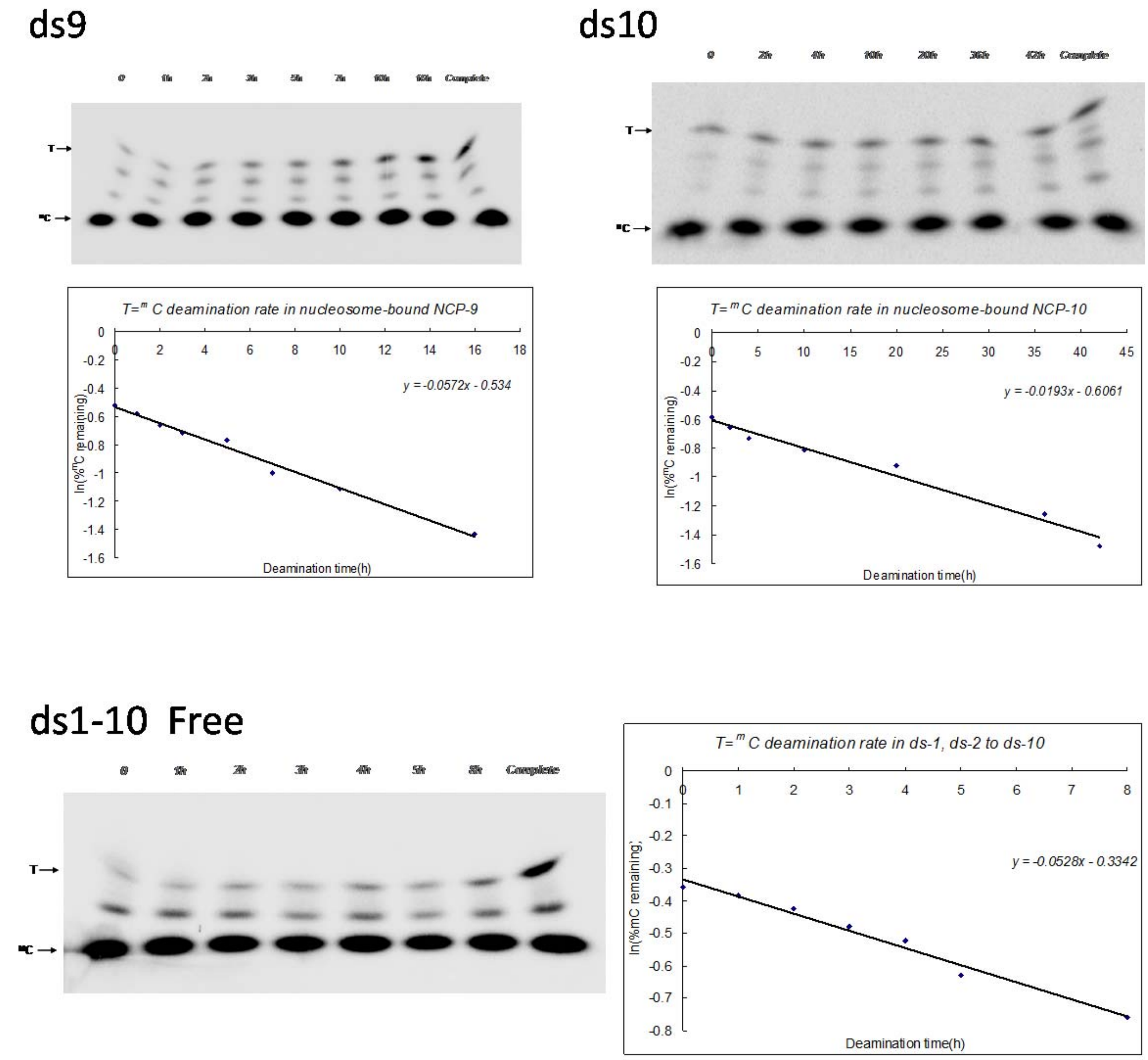

Figure S4 (cont.)

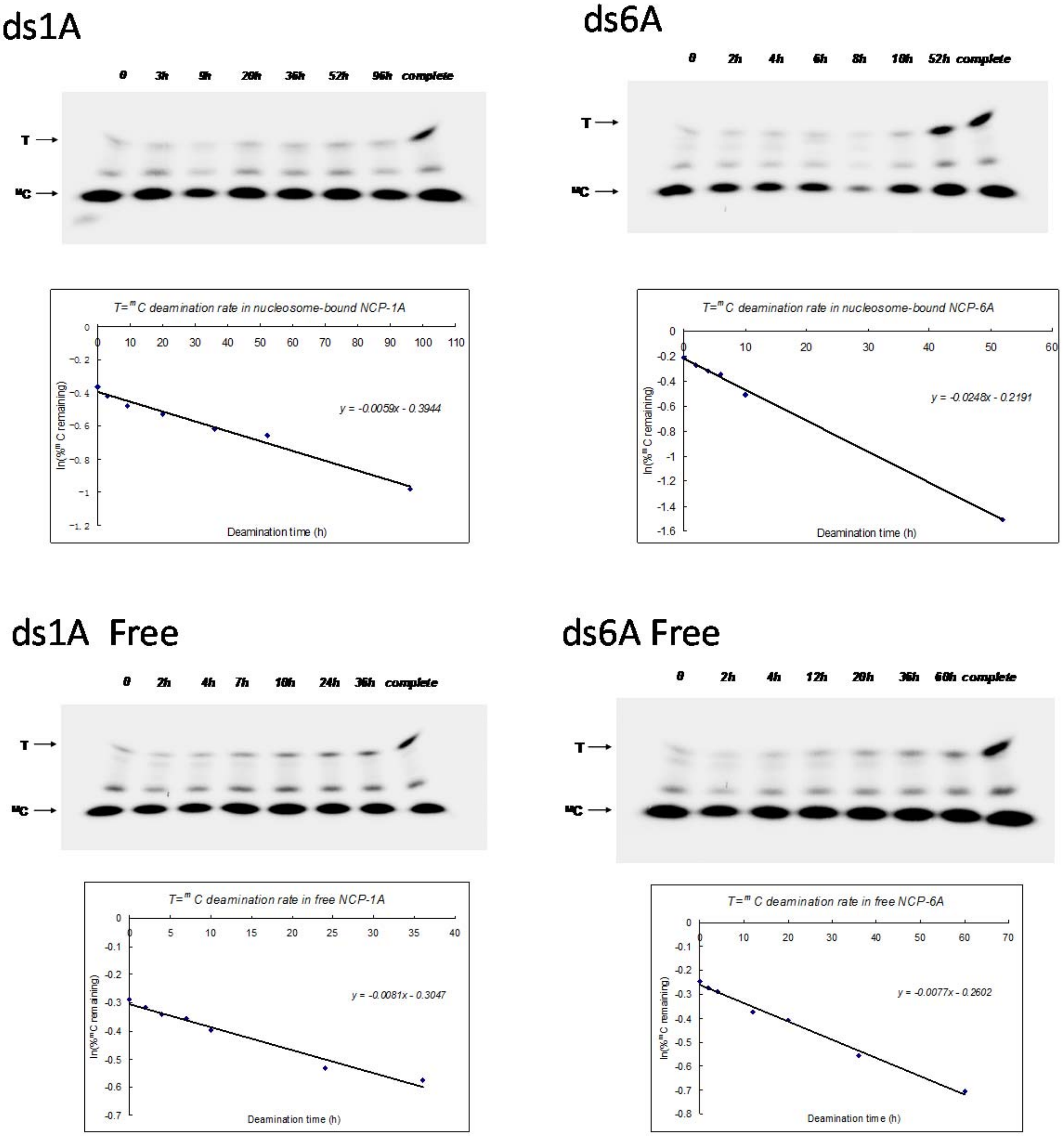

Figure S5. Deamination rate of T=^{m}CA CPD in nucleosome-bound and nucleosome-free ds1A & 6A . Gel electrophoresis of the NP degradation products of the photoreverted DNA as a function of deamination time. Plots of the log fraction ^{m}C in T=^{m}C remaining vs time.

A.

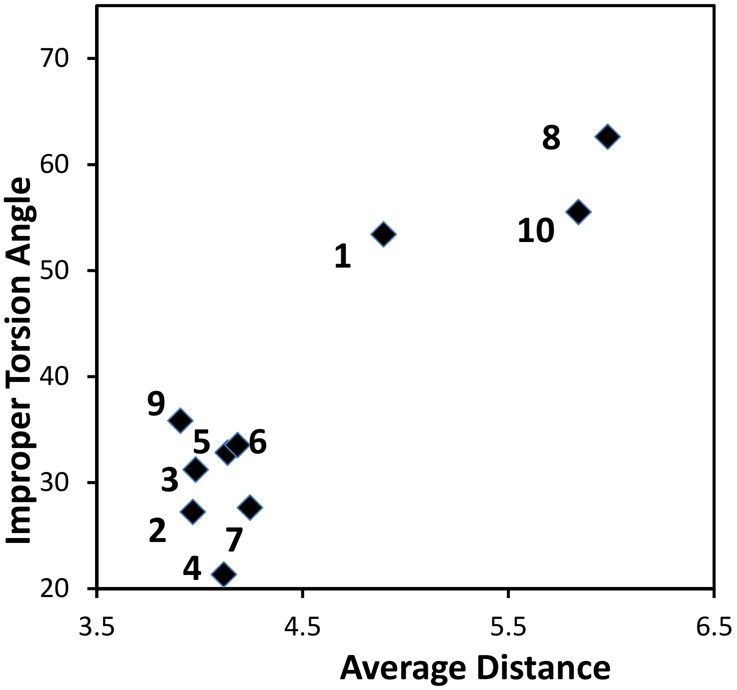

B.

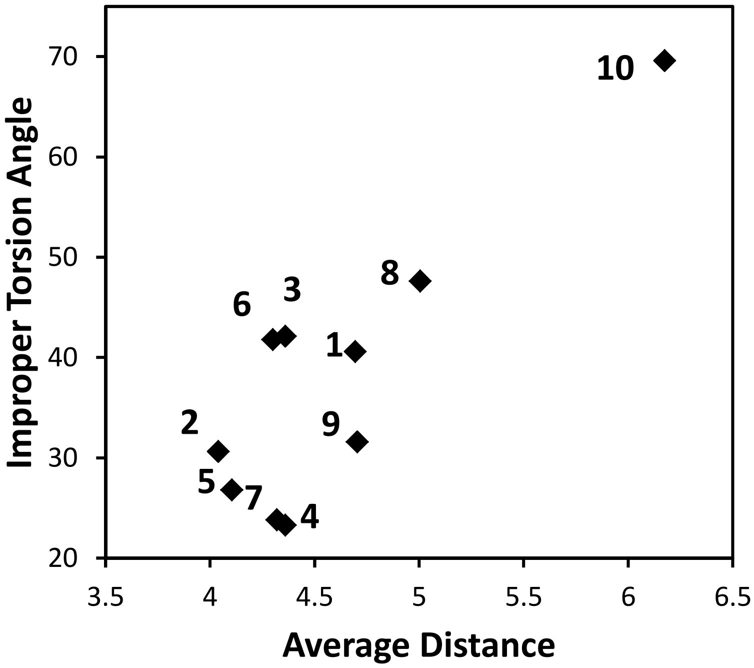

Figure S6. Relative orientation between adjacent C5-C6 bonds in T-tracts derived from crystal structures of nucleosome core particles. The bases from -5 to +6 in A) 1KX5.pdb and B) 3UT9.pdb were converted to T-tracts and the average distance between the centers of the C5-C6 bonds and improper torsion angle C6-C5-C5'-C6' of adjacent thymidines were plotted against each other.

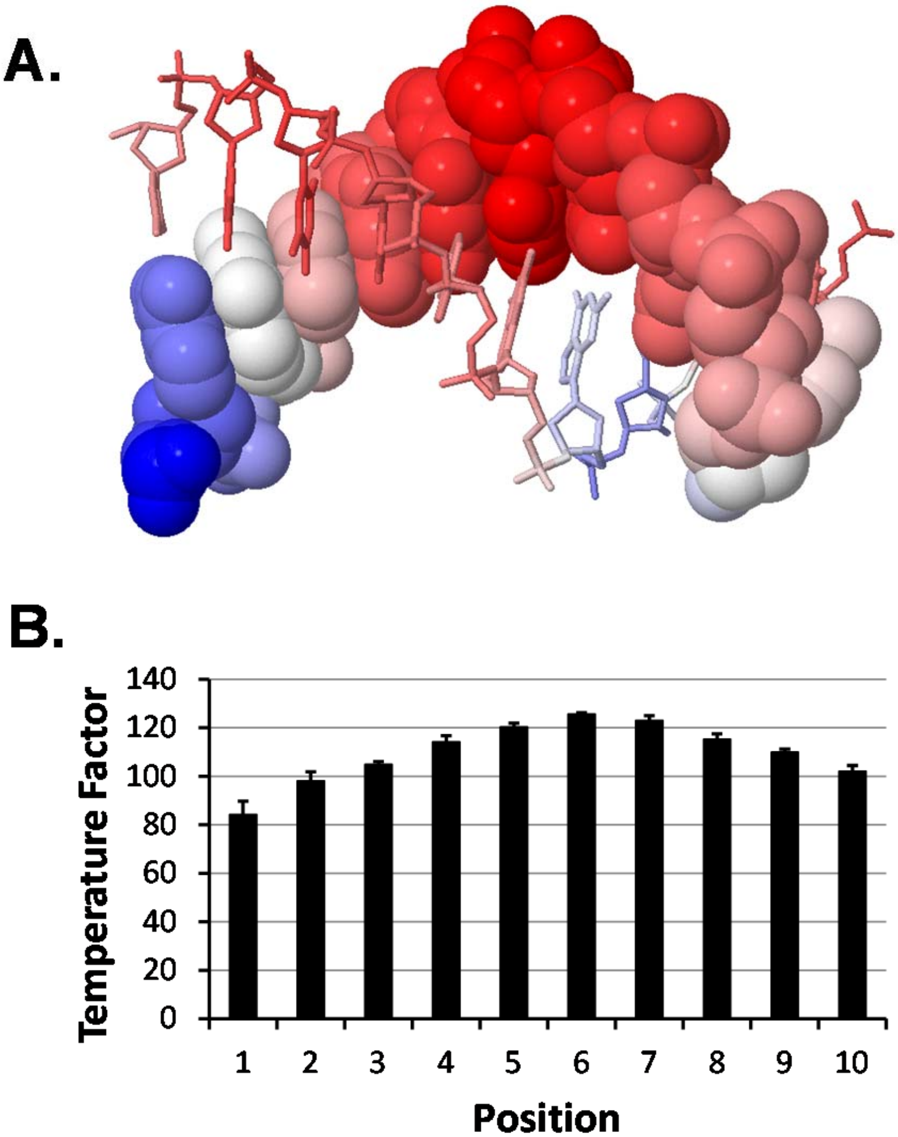

Figure S7. Temperature factors for the nucleotides in 3UT9.pbd corresponding to the ^{m}C positions in the current study. A) CPK image of the mC-containgin strand and wireframe image of the complementary strand for the nucleotides in 3UT9.pdb corresponding to the numbered ^{m}C sites in the present study. Blue corresponds to the lowest temperature factor and red to the highest temperature factor in the range. B) Histogram of the temperature factors for the averaged temperature factor for all atoms of a single nucleotide.
